# Titanium and Ruthenium Phthalocyanines for NO_2_ Sensors: A Mini-Review

**DOI:** 10.3390/s90705277

**Published:** 2009-07-06

**Authors:** Anna Maria Paoletti, Giovanna Pennesi, Gentilina Rossi, Amanda Generosi, Barbara Paci, Valerio Rossi Albertini

**Affiliations:** 1 CNR Istituto di Struttura della Materia, Sede di Montelibretti, P.B. 10, 00016 Monterotondo Stazione, Italy; E-Mails: gianna.pennesi@ism.cnr.it (G.P.); gentilina.rossi@ism.cnr.it (G.R.); 2 CNR Istituto di Struttura della Materia, Sede di TorVergata, Via del Fosso del Cavaliere, 100 - 00133 Rome, Italy; E-Mails: amanda.generosi@ism.cnr.it (A.G.), barbara.paci@ism.cnr.it (B.P.); valerio.rossi@ism.cnr.it (V.R.A.)

**Keywords:** phthalocyanine, X-ray reflectivity, NO_2_, sensors

## Abstract

This review presents studies devoted to the description and comprehension of phenomena connected with the sensing behaviour towards NO_2_ of films of two phthalocyanines, titanium *bis*-phthalocyanine and ruthenium phthalocyanine. Spectroscopic, conductometric, and morphological features recorded during exposure to the gas are explained and the mechanisms of gas-molecule interaction are also elucidated. The review also shows how X-ray reflectivity can be a useful tool for monitoring morphological parameters such as thickness and roughness that are demonstrated to be sensitive variables for monitoring the exposure of thin films of sensor materials to NO_2_ gas.

## 1. Introduction

Phthalocyanines (Pcs) [1,2] are organic compounds able to act as chemically sensitive films because of the various physical effects induced in them by their interaction with a large number of molecules. They have been widely used as thin-film semiconducting gas sensors for the detection of acceptor gases such as halogens or nitrogen oxides (NO_x_), as well as organic vapours.

**Figure 1.**
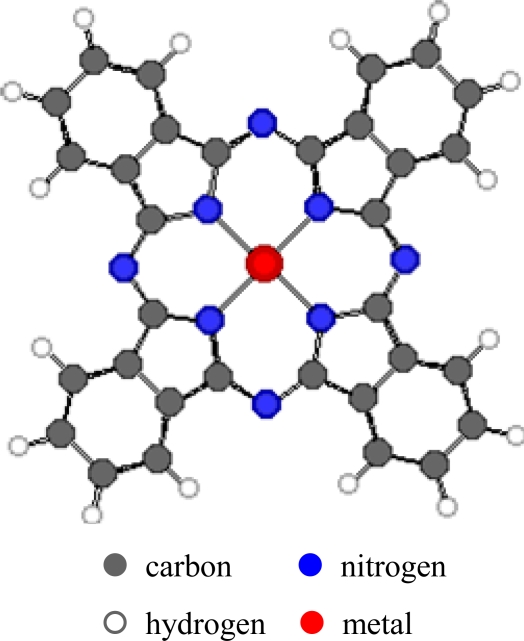
Structure of metal-phthalocyanines.

The properties of metal phthalocyanines (MPcs, Figure 1), particularly their redox properties, can be affected by the metal-ion coordination and by the peripheral attachment of additional atoms or groups that enhance or diminish the ionisation potential. Variation of the substituents in the side chains, the axial ligands or μ-bridging atom in the polynuclear derivatives can influence different detection properties of environmentally relevant gases. This can allow production of carefully designed and optimised thin films with different degrees of sensitivity, selectivity and stability. In addition, such films have high chemical and thermal stability in many environmental conditions. They can easily be produced as films by methods such as sublimation, spraying, Langmuir-Blodgett and spin-coating. The interactions between the Pc films and the gases may be irreversible chemical affinity, reversible (usually charge-transfer) chemical reaction or bulk sorption. These interactions result in detectable changes in physical properties of the films which include conductivity, mass and optical properties.

The electronic conductivity of MPcs changes in the presence of gases that are electron acceptors or donors, even at room temperature. Halogens (Cl_2_, Br_2_ and I_2_) [3–5] ozone [6] and nitrogen oxides [7–9] can be detected at the ppb level.

The increase of conductivity during exposure to oxidising gases can be explained as follows: the gas diffuses into the film, displaces other adsorbed species such as oxygen, and then a charge transfer complex is generated between the molecule and the acceptor gas and charge carriers (holes) are created in the film matrix [10]. These charge carriers are responsible for the increase of the conductivity. Nevertheless, the low conductivity of MPcs makes the electrical measurements difficult and the long recovery times needed to restore the original conductivity are another significant shortcoming.

Sandwich-type lanthanide *bis-*phthalocyanines can overcome these important problems because, since they are intrinsic semiconductors, changes in conductivity caused by small amounts of gases yield measurable signals, improving the performances of the sensor. The exposure of LnPc_2_ to oxidant gases such as NO_2_ causes drastic changes in the conductivity at room temperature [11–15], but the response is different compared with that of the transition metal phthalocyanines described above. First, the interaction produces an increase of the conductivity, then the conductivity undergoes a gradual decrease. The reason for this behaviour is that NO_2_ gradually transforms the intrinsic semiconductor LnPc_2_ into the oxidised form LnPc_2_^+^, causing a decrease of the conductivity after a long exposure [11].

The intensity and kinetics of the response to a particular gas are therefore related to the nature of the phthalocyanine compound used as the sensitive material. In addition, the charge transport process and the possibility of measuring a macroscopic conductance depend on the relative orientation and separation of adjacent molecules. The variation in conductivity is in fact controlled by the nature of the surface adsorption sites which is influenced by both the structure and morphology of the films and the latter varies considerably, depending on the method of deposition used. This is particularly evident in Langmuir–Blodgett films in which the suppression of crystallinity has the effect of enhancing the matrix permeability and improving the sensor performance [16].

Whit regards to the sublimated films, phthalocyanines crystallise as a variety of polymorphs, and the two main phases are identified as α and β. They are both characterised by a herringbone structure with the molecules stacked along the *b*-axis, but the polymorphs are differentiated by the angle between the plane of the molecule and the stacking axis. Sublimated films of transition metal phthalocyanines may have amorphous or α or β crystalline nature depending on sublimation conditions. Annealing of a sublimated crystalline film transforms the α̣ form to the β form and changes its electrical conduction and gas response characteristics [17]. This indicates that for sublimated films morphology also plays a fundamental role in the electrical response to gas exposure. Indeed, the morphology must accommodate both the charge transfer interaction and charge carrier transport. It is commonly accepted that the charge transfer interaction is easier if the face of a phthalocyanine ring rather than an edge is available, i.e. if the molecules are arranged face-on instead of edge-on with respect to the surface of substrates. On the other hand, charge carrier transport is facilitated by a long-range stacking of co-facially oriented phthalocyanine rings.

Amorphous films are prepared by vacuum sublimation (10^−6^ Torr) onto liquid-nitrogen-cooled (100K) substrates [18]. These films are stable at room temperature but crystallise to an α̣ phase on annealing over the temperature range 60–140 °C followed by an α̣ to β phase transition at more elevated temperature depending on the central metal [19]. The structure of sublimated films is important since, as we have already mentioned, it determines how gases may enter the lattice and interact with the phthalocyanine units. We can infer that structure and morphology are subjected to notable variations during the exposure and certain information can be obtained from these changes. It is therefore convenient to approach the study of the metal phthalocyanines - NO_2_ gas interaction from a morphological point of view as well.

Several researchers have studied the mechanism of gas diffusion in a thin/thick film sensor, especially with regard to semiconducting oxide thin films [20]. A diffusion-reaction model was found to interpret the various performances of gas-sensing devices satisfactorily, together with the changes in thickness, roughness, or crystallinity of the films [21,22]. Nevertheless, the reported measurements were focused on the devices’ electrical response, while the structural and morphological changes accompanying the exposure to the gas were not investigated [23]. An appropriate tool for evaluating the latter is X-ray reflectometry. This technique is commonly utilised to probe the properties of surfaces and interfaces of layered samples like films deposited on substrates, multilayers, superlattices, etc. [24]. In particular, the application of Energy Dispersive X-ray Reflectometry (EDXR) [25–27] allows a detailed study in situ of phthalocyanine thin films and of their morphological change (thickness and roughness) as a consequence of the exposure to NO_2_ gas flow.

In this review the investigations on evaporated films of two different types of non-monomeric phthalocyanine derivatives, a sandwich and a dimer, will be illustrated in terms of their potential exploitation as sensors for NO_2_. The two chosen compounds are singular regarding their chemical-physical properties: the titanium *bis*-phthalocyanine is a sandwich-type molecule with peculiar redox properties that reversibly change its colour from yellow to green to red; the ruthenium phthalocyanine is the only known dimeric unsubstituted phthalocyanine and its unique columnar arrangement makes it an intrinsic semiconductor. Their response to NO_2_ is different and the interaction between the gas and the molecule and electrical and optical behaviour have been analysed in relation to the morphological evolution of the films of both compounds during exposure. Furthermore, the reported studies also show that thickness and roughness are characteristics sensitive to exposure to NO_2_ gas and X-ray reflectivity can be a useful tool for monitoring these morphological parameters.

In the reviewed articles all the measurements performed to test the sensing behaviour were recorded in measure-chambers designed on purpose, at room temperature and ambient pressure; mass-flow controllers adjusted flow and concentration of the gases; more details can be found in the experimental sections of the related articles.

## 2. Titanium *bis*-Phthalocyanine

**Figure 2.**
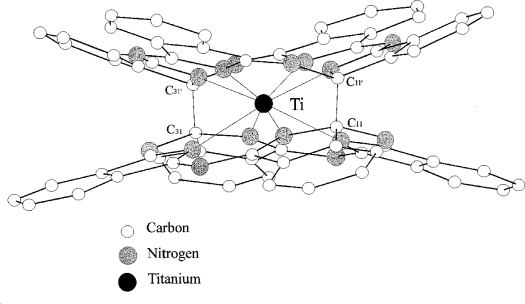
Side-view of titanium *bis*-phthalocyanine. Reproduced with the permission from reference [32].

Titanium *bis*-phthalocyanine “TiPc_2_” (Figure 2) [28] is a sandwich-type phthalocyanine with a single metal atom coordinating two macrocycles; unlike most other sandwich phthalocyanines it has two planes ‘stapled’ by two carbon-carbon bonds. The neutral complex shows an unusual yellow-orange colour because of the breaking of the π delocalisation by the two carbon σ bond. It is easily sublimed and the thin films obtained are microcrystalline and stable in air. The conductivity at RT is < 10^−12^ Ω^−1^ cm^−1^ and classifies TiPc_2_ as an insulator like other M(IV)Pc_2_ sandwich complexes and MPc monomeric compounds[1,2].

The peculiar redox properties of this compound have been extensively studied [29–31] because of the ability to break and restore reversibly the ‘stapling’ C-C bonds upon oxidation; the change in the electrical and optical properties, occurring during the oxidation, make it ideal for sensing and electrochromic applications.

### 2.1. Optical Behaviour during Exposure to NO_2_

**Figure 3.**
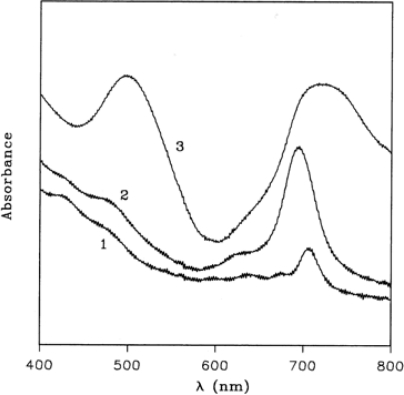
Spectra of TiPc_2_ film during oxidation by 100 ppm of NO_2_ gas in N_2_ at RT: (1) neutral yellow film; (2) after partial oxidation, green film; and (3) after complete oxidation, red film. Reproduced with the permission from reference [32].

Figure 3 (curve 1) shows the electronic absorption spectrum of TiPc_2_ film in the region 800–400 nm, the spectrum is lacking the characteristic absorption around 600–700 nm, known as Q band, owing to the dramatic modification of the chromophore mentioned above.

During the oxidation process, spectral changes are observed and two steps are recorded. In the first one (Figure 3, curve 2), a maximum at 695 nm appears after 1 h of exposure to 100 ppm of NO_2_ gas in N_2_, while further exposure produces the final spectrum with maxima at 718 and 498 nm (Figure 3, curve 3). Such spectral changes are analogous to those already observed in the electrochromic studies on this compound [30]. The electrochemical oxidation in fact produces monocation (TiPc_2_^+^) with a maximum at 710 nm and dication (TiPc_2_^++^) with two absorptions at 756 and 534 nm. In a similar way the NO_2_ gas promotes the formation of two oxidised species according to the following general equations:

(1) $$\left[{\mathrm{TiPc}}_{2}+{\mathrm{NO}}_{2}↔{\mathrm{TiPc}}_{2}^{+},{\mathrm{NO}}_{2}^{-}\right]$$

(2) $$\left[{\mathrm{TiPc}}_{2}^{+}+{\mathrm{NO}}_{2}↔{\mathrm{TiPc}}_{2}^{++},{\mathrm{NO}}_{2}^{-}\right]$$

### 2.2. Conductometric Behaviour during Exposure to NO_2_

**Figure 4.**
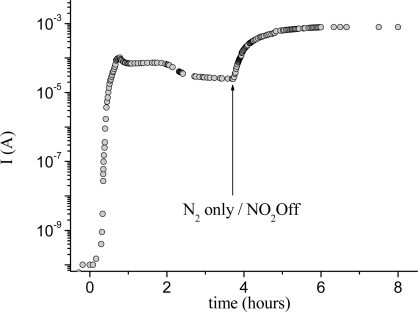
Variation of the current intensity of 50 nm thick TiPc_2_ film evaporated on an interdigitated electrode as a function of time when exposed to a mixture of NO_2_/N_2_ (20 nmol/s and 180 nmol/s, respectively) and then to pure N_2_. (a) Formation of the monocationic species (TiPc_2_^+^). (b) Equilibrium between the monocationic (TiPc_2_^+^) and bicationic (TiPc_2_^++^) species. (c) Introduction of pure N_2_. The y axis is represented in logarithmic scale for better appreciation of the trend of the curve. Reproduced with the permission from reference [33].

Along with the spectral variation occurring during the exposure to a constant concentration of nitrogen dioxide, the current variation of TiPc_2_ films shows an increase after about 15 min of exposure and then, passing through a maximum, a rapid decrease is observed; the removal of the NO_2_ leads to a new increase up to a higher maximum, after which a very slow decrease occurs. This behaviour is similar to that of other *bis*-phthalocyanines like LuPc_2_ but it is very different from the response of monomeric metal-phthalocyanines that normally display an increase of conductivity up to a constant final value [10]. Such a trend allows us to discriminate between the two oxidation processes occurring in the film. In Figure 4 it is possible to see the two distinct processes clearly.

This result can be explained by the mechanism already proposed for other sandwich-type phthalocyanines [11] according to which the conductivity response is attributed to the two chemical species (each one characterised by different conductivity and spectrum), as described below.

The two oxidised species, monocation and bication, are responsible for the conductivity behaviour. Although both species are present, the monocationic species has the main influence on the conduction process. With reference to Figure 4a, the first conductivity peak corresponds to the formation of the monocation species (TiPc_2_^+^) as confirmed also by the visible spectra. As the reaction proceeds, the equilibrium is shifted towards the second process and the dicationic species (TiPc_2_^++^), which is less conductive then the monocation, coexists with the first one, inducing a slight decrease of current (Figure 4b). This dynamic redox equilibrium, which takes place between the two cationic phthalocyanine species, corresponds in the conductivity curve to the plateau following the first peak. Finally, when the mobility of the charge carriers decreases because of the rise of the unfavourable sites to which the ‘hole’ can transfer, the conductivity decreases. At this point (Figure 4c), according to Equation (2), the removal of NO_2_ from the experimental chamber shifts the overall redox equilibrium back to Equation (1). As a consequence, a second conductivity maximum is reached, followed by a very slow decrease owing to the progressive diminution of conductive species.

### 2.3. Morphological Changes

**Figure 5.**
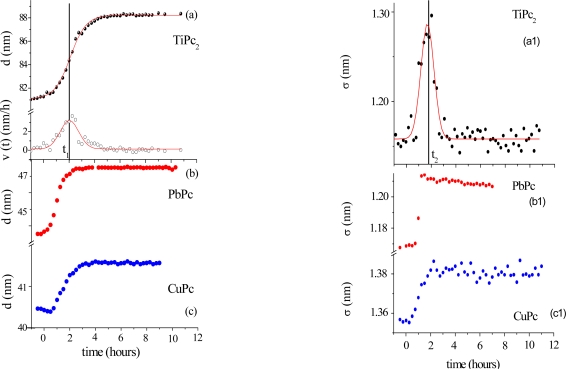
(a) Thickness evolution of an 80 nm thick TiPc_2_ film exposed to an NO_2_ gas flow of 20nmol/sec. and its derivative curve. In (b) and (c) the same time evolution is shown in the case of a PbPc and a CuPc film respectively. (a1) Simultaneous roughness evolution Roughness curves obtained for a PbPc and a CuPc film are shown in (b1) and (c1) respectively [38].

Morphological variations occurring during the gas exposure have been analysed by monitoring of two parameters, thickness, d and roughness, σ, derived from EDXR spectra recorded in situ. d and σ are proportional to the change of frequency of reflection curves and to the change in amplitude respectively, as extensively described in [33,34]. They are shown in Figure 5.

The expansion process, accounting for the d(t) trend, can be easily interpreted considering the d(t) derivative, namely the film growth speed v(t). As the gas starts flowing in the cell, the film bulk response is not immediate: at the beginning the d(t) profiles remain almost flat, so that v(t) is small (induction time of about 1 h). Then v(t) increases rapidly until a maximum value is reached. In the following step, the process slows down and, finally, v(t) approaches zero, indicating that the film thickening has concluded.

This film expansion, as expected, is similar to that observed in other metal-phthalocyanine films exposed to the same gas, and can be related to the bulk diffusion process. As shown in Figure 5b and c, when MPcs with M = Cu and Pb are considered, the thickness expansion follows the same kinetic behaviour, despite the different metal involved and the different film packing characteristics [35–37].

The roughness evolution of the same TiPc_2_ film (Figure 5a_1_), however, shows a completely different behaviour with respect to the other MPc considered (Figure 5b_1_ and 5c_1_).

The surface response usually consists of a monotonic increase of roughness during the first part of the exposure to the gas, followed by a plateau, when the saturation is reached, as can be seen in Figure 5b_1_ and c_1_, where the two σ(t) curves relative to the PbPc and the CuPc film are reported for comparison. While these follow a Boltzmann-like trend, the TiPc_2_ σ̣ (t) curve exhibits a peak. From Figure 5a and a_1_, it is evident that the time t_2_ at which the surface roughness reaches its maximum value coincides with the time t_1_ of maximum growth speed. This coincidence reproducibly occurs in all the samples investigated. Two different explanations have been proposed [34]: the first is the attribution of such behaviour to a thermodynamic effect, the second is the attribution to a kinetic effect.

In the case of a thermodynamic effect, the σ(t) peak represents the transformation from an initial arrangement of the layer formed by the NO_2_–TiPc_2_ molecules interacting at the film surface to a final arrangement, passing through an intermediate phase, that is, a transition from the original surface structure to a new one that exhibits a roughness comparable with the previous one.

Alternatively, a kinetic explanation can be provided. In this case, the increase of σ (t) would correspond to the random deposition of an increasing number of gas molecules that, by interacting with the film surface, form a disordered and rougher layer. Since the diffusion of the gas into the film takes a certain time, the ‘crowding’ of the molecules at the surface increases progressively as well as the roughness. When the molecules penetrate inside the film, the roughness returns approximately to its initial value.

To establish which of two hypotheses applies, the same kind of measurements have been carried out at increasing concentrations of NO_2_. The roughness curves reach about the same maximum value independently of the gas concentration indicating that the phenomenon is a thermodynamic effect and corresponds to a surface morphological rearrangement of the TiPc_2_ films [34].

**Figure 6.**
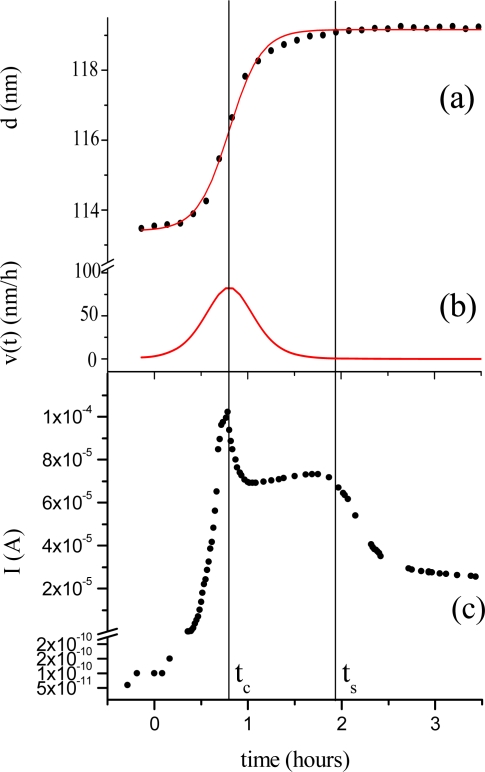
(a) Evolution of the parameter d of a 110 nm thick TiPc_2_ film as a function of time when exposed to an NO_2_ 50 ppm gas flow. (b) Derivative of d vs t. (c) Evolution of the current as a function of time as in (a) [33].

Morphological parameters correlate also with the electrical response of the TiPc_2_ films when exposed to an NO_2_ gas [33]. In Figure 6 the thickness evolution (a) is plotted as a function of time together with the current intensity change (c). The diffusion of the gas (50 ppm, 20 nmol/sec in 180 nmol/sec N_2_) into the bulk of the film reaches the maximum of velocity at characteristic time t_c_ = 0.8 hours (b). The conductivity peak, corresponding to the formation of the monocation species (TiPc_2_^+^), coincides with the maximum velocity of the diffusion process (c), while the conductivity drop corresponds to the saturation time of the diffusion of the gas molecules into the bulk t_s_.

### 2.4. Conductometric and Optical Sensor Performances

To test the new material for gas-sensing applications, different features have been taken into account: performances of conductivity variation and of optical absorption as a function of NO_2_ concentration, reversibility and, from a morphological perspective, selectivity.

Tests of conductivity were performed [32] showing that in the presence of lower gas concentrations the maxima of conductivity were reached after longer periods and the response time was entirely unsatisfactory, complete reversibility of the system was proved to occur even at RT under vacuum only after long periods and in 15 min at 100 °C.

**Figure 7.**
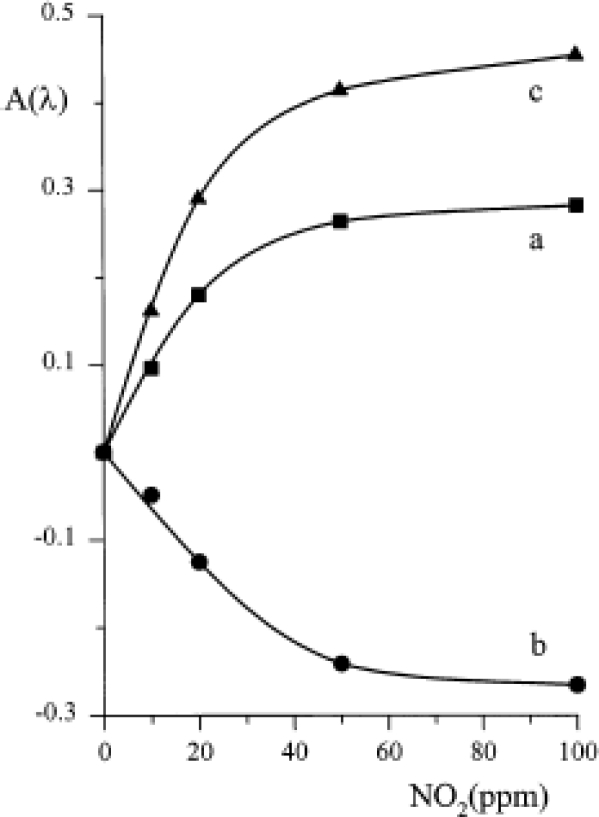
Absorbance versus concentration in correspondence with the three absorption peaks of the immobilised TiPc_2_ (curve a: λ 506 nm; curve b: λ 675 nm; curve c: λ 720 nm). Reproduced with the permission from reference [39].

Conversely, the original optical behaviour was exploited for designing an optical sensor. A suitable quantity of TiPc_2_ was immobilised by evaporation or casting on a plastic reflector (optode) placed in front of an optical fibre, as comprehensively explained in [39]. For each of the three maxima shown in spectra 2 and 3 of Figure 3 the variation of absorbance as a function of NO_2_ concentration was recorded (Figure 7); a linearity up to 20 ppm of NO_2_ was demonstrated and a limit of detection of about 1 ppm was feasible.

**Figure 8.**
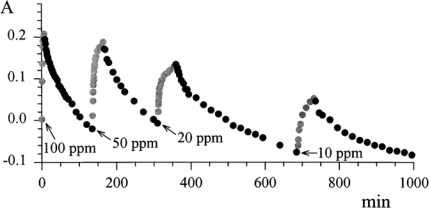
Absorbance versus time for λ 720 nm for different cycles of NO_2_-N_2_, and vice-versa of TiPc_2,_ immobilised on the plastic reflector (the ‘optode’) exposed to different concentrations of NO_2_ in correspondence with the arrows. Reproduced with the permission from reference [39].

The reversibility of the process was investigated by exposing the ‘optode’ to many cycles of N_2_-NO_2_, and vice versa. Figure 8 shows the absorbance at λ 720 nm as a function of time in the presence of different exposures to gas mixtures.

The results are summarised in Table 1, in which the response time (*τ* 90%) and the absorbance steps are reported, when there is a change in the concentration of NO_2_ from 0 to 100 ppm and vice versa.

As expected, the ‘optode’ built by casting was characterised by a better sensitivity, since the available surface for the immobilisation of the chemical transducer was larger than that for the evaporation process. On the other hand, the sublimated layer was characterised by a faster response time. From these promising results, a better optical sensor device was designed [40].

Given that the kinetics associated with equilibria (1) and (2) is completely different, since the first one is much faster than the second, and that both oxidations are reversible, but with completely different time-constants, i.e. days for the first one, minutes for the second one, the attention was focused on equilibrium (1). Light-emitting diodes were used as narrow-band sources in order to diminish the level of the light that impinges on the sensing compound, and to eliminate any possible problems of photodecomposition.

A different immobilisation of TiPc_2_ was carried out, in order to improve the sensitivity and the reversibility of the probe. The utilisation of an alumina membrane with a porosity of 0.2 μm made it possible to have a very large active surface and consequently a very effective interaction with gas. A suitable optoelectronic unit was developed for the characterisation of the ‘optode’. Since the reversibility of equilibrium (1) is very slow, the reduction of the monocationic species (TiPc_2_^+^) was accelerated by exposing the ‘optode’ to the UV light with a procedure that was the object of an international patent [41]. The exposure to UV light of the oxidized ‘optode’ yields a complete recovery of the initial state. Close examination, performed with a quadruple mass spectroscopy on the desorbed molecules, suggests that the UV irradiation causes the breaking of the charge-transfer bond between the NO_2_ and the TiPc_2_ in the compound (TiPc_2_^+^ NO_2_^−^) so the release of NO_2_ can be detected. With this new ‘optode’, a fully reversible system and a detection limit of 0.6 ppm were obtained.

Also the selectivity of the TiPc_2_ was found to be extremely high with regard to nitrogen dioxide, when compared with other phthalocyanines [10] or with other chemical transducers for NO_2_ detection [42,43], which are generally characterised by many cross-sensitivities. In fact, no interferences from SO_2_, CO, ambient air, NH_3_, or NO were observed. In particular, the absence of interaction with NO is noteworthy.

The reversibility of the system was also followed by monitoring the morphology recovery by means of EDXR spectroscopy. A series of TiPc_2_ evaporated films were exposed to an NO_2_ gas flow and then they underwent different thermal treatments (130 and 200 °C) under vacuum and UV light exposure at RT; all films were found to return to their original thickness. The same behaviour was observed for all samples studied, showing that the chemical reversibility of the sensor is also related to the recovery of the initial film thickness [38].

## 3. Ruthenium Phthalocyanine

Ruthenium phthalocyanine [44] is a very interesting compound because of its many unusual properties. It is obtained as an amorphous powder from thermal treatment of ruthenium phthalocyanine bis-adducts such as [PcRu(quinoline)_2_], [PcRu(pyridine)_2_] [45] or [PcRu(DMSO)_2_] [46] and its structure, examined first by the large-angle X-ray scattering (LAXS) [47] technique, was resolved only if a dimeric molecule was taken into account. This unique structure was also confirmed by Extended X-ray Absorption Fine Structure (EXAFS)[48] and explains the paramagnetic behaviour of (PcRu)_2_ with a room-temperature magnetic moment (2.54 *μ*_B_), which is strongly temperature-dependent in the range 300–306 K. Interpretation of the magnetic behaviour leads to an electronic energy level diagram which locates the highest energy electrons for the dimer in the orbital sequence σ^2^π^4^δ^2^δ^*2^π^*2^ (in the Molecular Orbital approach), consistent with the presence of a plain Ru(II)-Ru(II) double bond [47].

The bulk structure consists of dimeric units held together by a Ru-Ru bond (2.40 Å) and superimposed on each other to give an aggregate of formula [(PcRu)_2_]_n_ (average n = 6), with the ‘examers’ randomly arranged in the solid amorphous material [47].

The complex is stable in air as a solid material, but readily interacts with dioxygen in solution, and its catalytic dioxygen activation and oxygen atom transfer towards olefins have been studied [47].

In contrast to other non-radical phthalocyanines, that when undoped are insulating materials (σ_RT_ < 10^−12^ Ω^−1^ cm^−1^), the ruthenium phthalocyanine is an intrinsic semiconductor (σ_RT_ = 1 × 10^−5^ Ω^−1^ cm^−1^) [47]. The semiconducting properties of the material have been attributed to the π-π interaction between adjacent macrocycles within the stacked assemblies formed by the dimeric units. The participation of the Ru centres apparently has to be excluded on the basis of the ‘internal’ location within the dimer of the strongly interacting Ru couples and the long interdimer Ru-Ru contacts (4.32 Å).

The dimeric structure and the stacked arrangement are preserved in the evaporated films [23]. X-ray data indicate that in the passage from the bulk to the film the only change is the different length of columnar packing of dimeric molecules, more wide-ranging in the film than in the bulk material. Ten dimers are in fact arranged along the stacking direction (parallel to the Ru-Ru bonds). The higher order observed was also supported by conductivity measurements, the arrangement in a one-dimensional stacking being responsible for the improvement of the electrical properties: σ_RT_ = 1 × 10^−4^ Ω^−1^ cm^−1^, one order of magnitude greater than in the powder.

The involvement of the ruthenium in the reactivity of (RuPc)_2_ towards di-oxygen has been demonstrated not only by the catalytic properties, but also from the interaction with molecular oxygen in solution in different media that yields a solid amorphous oxygen-containing material of general formula HO-[(Pc)Ru^IV^O]*n*-H [49]. This polymer, like (PcRu)_2_, is also an electrically conductive material; its measured *σ*_RT_ value is 1.10^−2^ Ω^−1^ cm^−1^, that is much higher (10^3^ times) than that of (PcRu)_2_. In (PcRu)_2_ the charge transfer along the stacked material occurs by *π-π* interactions of adjacent, though domed, Pc rings. Vice versa, in the polymer, the role played by the central metal is certainly relevant, since the Ru(IV) ions are located in the centre of the planar Pc units and are joined by Ru-O-Ru bridges, thus allowing charge transfer to take place through a d*π*(Ru)-p*π*(O)-d*π*(Ru) linkage.

The reactivity of (PcRu)_2_ towards ‘small molecules’, whether it involves both macrocycles and central metals or not, has also been studied for possible sensing applications. Unfortunately, the performance of (PcRu)_2_ films with regard to NO_2_ (0–100 ppm) yielded poor results in terms of reversibility, even at very low concentrations [50]. Therefore, we addressed our study to a deep and detailed comprehension of the interaction mechanism between (PcRu)_2_ film and NO_2_ gas, with the aim of establishing the role of the two Ru atoms and the two macrocycle rings, both factors being closely connected with the performance and reversibility of the system. The supramolecular aggregation could certainly affect the gas diffusion [51], and furthermore it should be taken into account that the interaction with the NO_2_ molecule could lead even to significant molecular rearrangement. Structural investigation by Extended X-ray Absorption Fine Structure (EXAFS) technique and surface characterisation by X-ray Photoelectron Spectroscopy (XPS), joined to UV-Visible spectra, recorded in situ during the gas exposure, have allowed a complete interpretation of the phenomena involved [52]. Moreover, several in-depth studies have also been published in the last few years on evolution of the morphological parameters (thickness and roughness) already mentioned, also joined to conductometric variations, and they have provided further understanding of the effect of structural and morphological features on the sensing behaviour of (PcRu)_2_ [51,53–57].

### 3.1. Spectroscopic Features of (PcRu)_2_ Films during Exposure to NO_2_

**Figure 10.**
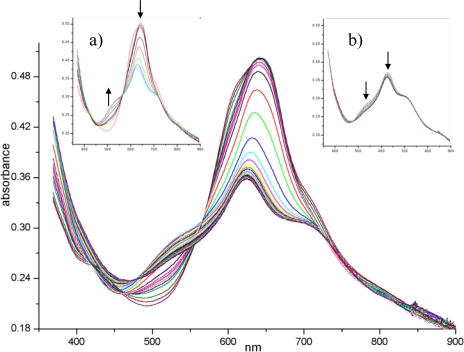
UV-Visible spectra of a 90 nm thick (PcRu)_2_ film recorded during exposure to 92 ppm of NO_2_ gas in dry air; inset a) first 90 min of exposure; inset b) from 90 to 280 min of exposure.

Figure 10 shows the complete evolution of the visible spectrum of the (PcRu)_2_ film during NO_2_ exposure. The initial spectrum of the evaporated material displays the maximum of Q band at 630 nm with a shoulder at 608 nm and a less evident one at 710 nm: a detailed interpretation of this kind of spectrum can be found in [58]. The most dramatic changes in the spectrum occur during the first 90 min (Figure 10 inset a). After a few minutes of NO_2_ gas diffusion, the shoulders around 608 and 430 nm disappear.Since these small absorptions are attributable to the intra-dimer interaction in the solid state [58], the penetration of NO_2_ into the core of the stacks separates the dimeric units, disarranges the columnar order and gives rise to a more disordered arrangement. The overall intensity decreases while a new peak at 510 nm arises, these spectral features being characteristic of an oxidised radical form of the phthalocyanine complex, centred on the macrocycle, as widely recognised [59,60]. In addition, XPS and EXAFS confirm that the Ru metal is simultaneously involved in the process through its link with NO_2_ molecule [52]. The second step of the process (Figure 10 inset b), that takes a longer period of gas exposure up to 280 min, shows a slow regression of the new 510 nm band that can be explained in terms of depletion of radical population owing to the radical coupling arising from the extensive oxidising condition. Finally, the forced desorption (under vacuum and/or by thermal treatments) of gas from the lattice almost restores the original spectrum but the shoulders at 430 and 608 nm are no longer recovered, as expected for structural modifications induced in the layer.

### 3.2. Conductivity and Morphological Response of (PcRu)_2_ during NO_2_ Exposure

The monitoring of thickness and roughness, performed by EDXR, of (PcRu)_2_ during the exposure confirms that the interaction with NO_2_ is a complex phenomenon. In fact, it is evident that the gas absorption process develops in two stages: a ‘surface’ adsorption process, probably in relationship with the ruthenium involvement, and a ‘bulk’ absorption process. The evolution of thickness follows two distinct trends: a rapid increase, as the gas starts flowing in the cell, followed by a slower growth up to a final plateau [51]. The observation of the surface roughness is further confirmation of such a two-step model. Indeed, the surface roughness increases in the same time interval in which the first step of thickness occurs, indicating that it is associated with the gas-surface interaction. Furthermore, the ‘surface’ adsorption process (first step) is irreversible (from a morphological perspective), and conversely the ‘bulk’ absorption process (second step) is reversible upon thermal reset at 130 °C. The first step can reasonably be attributed to the interaction between the ruthenium metal and the NO_2_ molecules and the second one is attributable to the diffusion of gas molecules in the film bulk [55].

**Figure 11.**
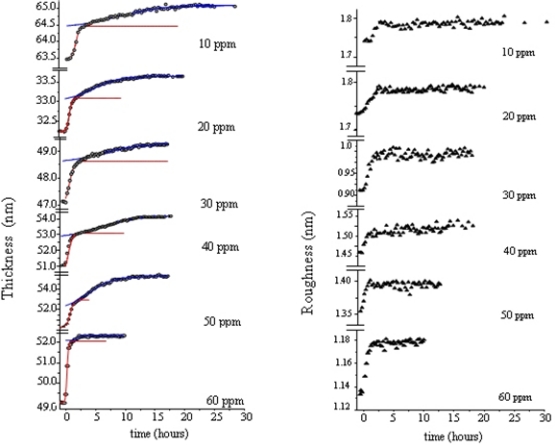
Time evolution of the morphological parameter d (thickness) and σ (roughness) for different NO_2_ concentrations [38] of (PcRu)_2_ films, evaporated on silicon substrates, of different initial thickness.

The slowdown occurring as the concentration decreases allows us easily to observe the characteristic two-step behaviour. In Figure 11 the time evolution of the thickness d is shown for different NO_2_ concentrations. The sigmoidal fit of the two distinct regions allows us to assess the characteristic times of the interactions. The right side of Figure 11 shows the time evolution of the roughness σ̣. It is independent of the different NO_2_ concentrations and reaches the maximum value along with the first step of the thickness evolution.

**Figure 12.**
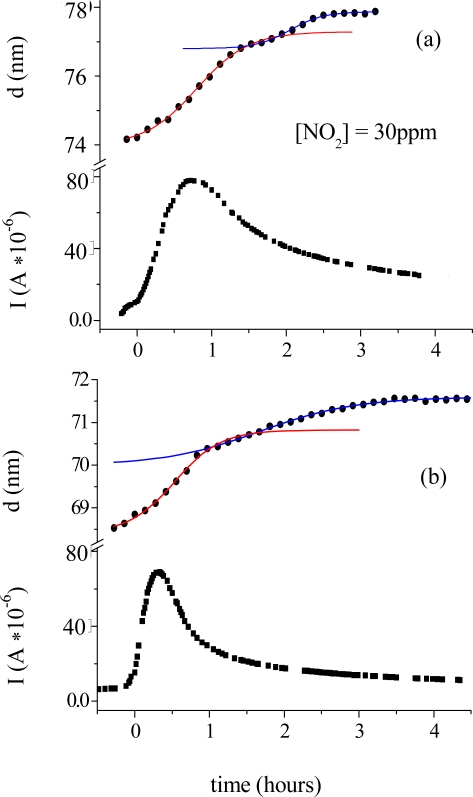
Time evolution of the morphological parameter d of (PcRu)_2_ films together with current change when exposed to a 30 ppm NO_2_ gas flow (a) and to a 50 ppm NO_2_ gas flow(b) [38].

Because for ruthenium phthalocyanine the study of morphological parameters also clearly shows that the phenomena connected with NO_2_ intake are both surface and bulk phenomena and because the previous studies such as visible spectroscopy and XPS show that the central metal is involved in the interaction then the study of electrical response could be meaningful. Figure 12 shows the preliminary results. It is evident that the time of response of current variation depends on NO_2_ concentration and that the overall conductivity change is comparable for the two samples independent of the NO_2_ concentration. The shape of d(t) is confirmed to be formed by two regions, the first one of which represents the surface interaction and is independent of the NO_2_ concentration. The different timing of the two functions does not allow us to correlate the surface interaction with the current trend, the latter being more probably related to the bulk diffusion. Moreover, the trend of current is similar to that displayed by titanium and other sandwich-type phthalocyanines. The drop of conductivity in such systems was attributed to the decrease of mobility of charge-carriers owing to the increase of dicationic species. In this case this kind of behaviour might shed light on the chemical reaction occurring between the NO_2_ molecule and the ruthenium phthalocyanine dimer. The involvement of the metal could in fact yields The involvement of the metal could in fact yield two possibilities: either the gas breaks the dimer and attaches the metal from both sides or it does not break the dimer and it attaches the dimer from both ends. If the latter is the case, then the behaviour of current is properly justified. In any case, further experiments need to be carried out to address this attribution properly.

The change of morphological parameters and the reversibility of these changes in relation to thermal treatments of (PcRu)_2_ films have also been investigated in a recent paper [56], in which are reported the effects of thermal treatments (pre- or post-exposure annealing at different temperature) and of the second cycle of exposure to NO_2_. For this purpose, EDXR and Atomic Force Microscopy (AFM) have been used together in situ during the exposure. Considering the variation of thickness and roughness as a sensitive signal of sensing capacity, the results of combined EDXR-AFM confirmed as expected, that morphological changes, induced by high temperatures, strongly affect the material sensing ability up to complete deactivation. Nevertheless it is possible to stabilise the film morphology by a pre-exposure thermal treatment that enhances the sensing performance in terms of response times and more resistance to subsequent thermal stress. These indications have to be validated in terms of conductivity performances and current, reflectivity and AFM measurements carried out simultaneously.

## 4. Conclusions

Phthalocyanines are well known materials for sensor devices and besides the commercial products, other uncommon compounds can be favourably utilised like titanium and ruthenium phthalocyanines. Mechanisms involved in the response need to be investigated in depth to obtain better performances, as clearly demonstrated, for instance, by the optical sensor designed for TiPc_2_ in which the discrimination between different equilibria has improved the limit of detection.

Investigation of such mechanisms generally takes into account spectral and electrical features, but also considers structural and morphological modifications. For these latter features, EDXR can be considered a sort of ‘morphological monitor’ and can offer a very sensitive tool for evaluating the response of novel gas-sensing materials. The results reviewed validate the use of the in situ EDXR technique as a powerful non-destructive tool to investigate the response of thickness and roughness of sensing films. At the same time it can provide useful fundamental information on gas-film interaction and it can be exploited to obtain data helpful during the work of the device, being sensitive to reset treatments and to gas concentrations in a range comparable to that of the usual electro-resistive and optical measurements.

## Figures and Tables

**Figure 9.**
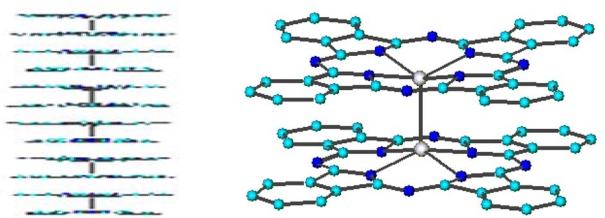
Sketch of the dimeric (PcRu)_2_ and its columnar arrangement.

**Table 1.** Response time (*τ* 90%) of the two ‘optodes’ for the step 0-100 ppm of NO_2_ and vice versa. Reproduced with the permission from reference [39].

	**0–100 ppm of NO_2_ and vice versa**								
	**λ 500 nm**			**λ 691 nm**			**λ 725 nm**		
	**ΔA**	**τ** (min)		**ΔA**	**τ** (min)		**ΔA**	**τ** (min)	
**immobilisation procedure**		0→100 ppm	100→0 ppm		0→100 ppm	100→0 ppm		0→100 ppm	100→0 ppm
evaporation casting	0.076 0.241	22 45	40–45 117	0.065 0.195	20 43	22 63	0.074 0.28	27 38	45–55 122
